# Number Size Distribution of Ambient Particles in a Typical Urban Site: The First Polish Assessment Based on Long-Term (9 Months) Measurements

**DOI:** 10.1155/2013/539568

**Published:** 2013-10-27

**Authors:** Krzysztof Klejnowski, Andrzej Krasa, Wioletta Rogula-Kozłowska, Barbara Błaszczak

**Affiliations:** Institute of Environmental Engineering of the Polish Academy of Sciences, ul. Skłodowskiej Curie 34, 41-819 Zabrze, Poland

## Abstract

This work presents results from the long-term measurements of particle number carried out at an urban background station in Zabrze, Poland. Ambient particles with aerodynamic diameters of between 28 nm and 10 **μ**m were investigated by means of a DEKATI thirteen-stage electrical low pressure impactor (ELPI). The particle number-size distribution was bimodal, whilst its density function had the local maxima in the aerodynamic diameter intervals 0.056–0.095 **μ**m and 0.157–0.263 **μ**m. The average particle number in winter was nearly twice as high as in summer. The greatest number concentrations in winter were those of the particles with diameters of between 0.617 and 2.41 **μ**m, that is, the anthropogenic particles from fossil fuel combustion. Approximately 99% of the particles observed in Zabrze had aerodynamic diameters ≤1 **μ**m—they may have originated from the combustion of biomass, liquid, and gaseous fuels in domestic stoves or in car engines. The daily variation of particle number was similar for both seasons—the highest values were observed in the morning (traffic rush hour) and in the afternoon/late evening (traffic and house heating emissions). An additional maximum (0.028–0.056 **μ**m) observed in the early afternoon in summer was due to the intensive formation of new PM particles from gas precursors.

## 1. Introduction

In order to assess the influence of particulate matter (PM) on the air quality, ecosystems, human health, and climate changes, it is necessary to be aware of its chemical composition and size distribution [1–3]. As humans are the most important recipients of environmental pollutants, differences in relationships between specific PM fractions (their concentrations) and morbidity and mortality of the human population must be taken into consideration.

There is no concentration threshold for PM in the atmospheric air, below which the PM impact on the human health could be ignored [4]. The correlations between suspended particles and health effects, including mortality, have been discovered for increasingly lower concentration levels [5, 6]. It is not clear which factors (i.e., PM mass concentration, number concentration, biological or chemical composition [7], physical properties, mass burden, particle number, total area, or electrostatic characteristics [8]) have the most crucial influence on human health. Nevertheless, the population exposed to PM always demonstrates adverse health effects.

Particles with aerodynamic diameters of between 10^−3^ and 100 *μ*m can occur everywhere in the ambient air. The number of particles with specific size present at a given site depends on many factors. These include the origin of PM at the discussed site [9], atmospheric processes (condensation, nucleation, and evaporation), chemical transformations, deposition, and removal with precipitation. It should be mentioned that particles with a diameter smaller than 100 nm, known as ultrafine particles, dominate the number concentrations but do make a small contribution to total aerosol particle mass [10, 11]. They represent excess health risks relative to fine (*d* < 2.5 *μ*m) or coarse particles (10 *μ*m < *d* < 2.5 *μ*m) of identical or similar chemical composition [12].

It is increasingly recognised that ultrafine particles can have significant implications on public health in addition to mass concentrations of particulate matter [10–12]. This is because ultrafine particles can easily be inhaled and deposited in the deeper regions of the respiratory tracts and have a higher surface area per unit volume than larger particles, thus increasing their capability to adsorb organic compounds, some of which are potentially carcinogenic [13]. Current legislation in Europe [14] requires mass concentration measurements of the PM_10_ and PM_2.5_ (ambient particles with aerodynamic diameter ≤ 10 and 2.5 *μ*m, resp.), whereas particle number concentration (including ultrafine particles) and size distribution are not routinely measured in monitoring networks [13, 15].

A number of studies described number concentration of PM in cities and urban surroundings [16–26]. It is clear that traffic is the most important source of ultrafine particles [17, 27, 28]. Emissions from gasoline- and diesel-fuelled vehicles alone can contribute to up to approximately 90% of the total particle number concentrations [29]. Kumar et al. [16] reports a summary of recently published studies on atmospheric nanoparticles in European cities. This covers a total of about 45 sampling locations in 30 different cities within 15 European countries for quantifying levels of roadside and urban background particle number concentrations (PNCs). Average PNCs at the reviewed roadside and urban background sites were found to be 3.82 ± 3.25 × 10^4^ and 1.63 ± 0.82 × 10^4^ cm^−3^, respectively, giving a roadside to background PNC ratio of ~2.4.

Biomass burning in local sources and nucleation processes significantly influence the particle number. Number concentrations are also affected by meteorological conditions [12]. Furthermore, PM concentrations and PM size distribution vary considerably in time and space [30]. For example, concentrations of nanoparticles can vary up to five or more orders of magnitude (from 10^2^ to 10^7^ cm^−3^) depending on environmental conditions and source strengths [15]. For these reasons, continuous PM measurements performed at many various sites, particularly in densely populated urban areas subject to high PM levels, seem extremely useful and practical.

Studies conducted in recent years prove that PM concentrations in Poland are often high or extremely high [31–33]. This fact, combined with the unclear character of number size distribution and the PM number concentration in the air, highlights the necessity to conduct continuous measurements in urban areas in Poland.

**Figure 1:**
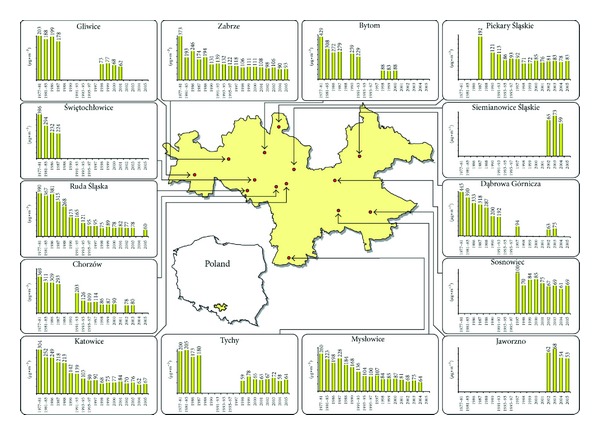
Total PM concentrations (*μ*g/m^3^) in 14 cities of the Upper Silesian urban area in the years 1977–2005 (figure taken from study [31]).

In terms of air protection, the Upper Silesian urban area is one of the most interesting regions, both in Poland and in Europe. Almost all industries (electrical, chemical, glass-making, textile, clothing and ceramic ones, ferrous and nonferrous metallurgy, machine building, hard coal mining, and coking) have been actively taking part in the deterioration of the natural environment for nearly 200 years. On the other hand, the Upper Silesian urban area experienced the largest national decrease in industrial air pollution brought about by the last three decades of economic transformations in Poland (e.g., the yearly dust fall in Zabrze exceeded 2100 g/m^2^ in the 1970s, oscillated between 700 and 800 g/m^2^ in the 1980s, and was lower than 350 g/m^2^ after the year 1995 [34]). Consequently, the PM concentration dropped significantly (Figure 1; see [31]). It is worth mentioning that Upper Silesia is also affected by periodically occurring episodes of very high concentrations of air pollutants (especially PM_2.5_ and PM_10_ concentrations in city centers in winter [35]), which increase the yearly PM concentrations.

The aim of the following study was to examine the number concentration and number size distribution of 13 PM fractions. The results discussed below come from the first long-term measurements (9 months) of particle number carried out in Poland.

## 2. Apparatus and Measurement Site

**Figure 2:**
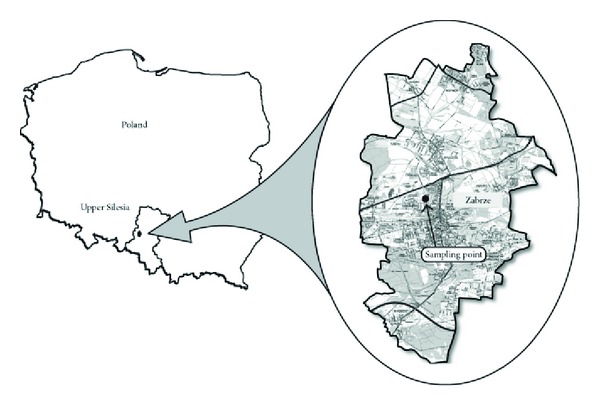
Measurement point location (figure taken from study [36]).

The measurement site selected for the experiment was located in Zabrze (Figure 2). It was representative for the air pollution conditions typical for the Upper Silesia urban area. Thus, it meets the requirements for the urban background site imposed by the Directive 2008/50/EC of the European Parliament and the Council [14]. Additionally, the impact of the industrial and municipal emissions on the Upper Silesia residential area was represented at this site and could be properly observed. Detailed description of measurement site surroundings was given elsewhere [34, 35].

Zabrze is one of the fourteen cities that form the Upper Silesia urban area. The area is located in the center of the Silesia province, Poland and occupies 141,230 km^2^. Its population is approximately 2.1 million (1,691 inhabitants per 1 km^2^). It is one of the most urbanized and industrialized regions of Central Europe. About 50% of the Silesia province gross product and 7% of the national GDP come from the Upper Silesian urban area. Six European capitals (Berlin, Prague, Vienna, Bratislava, Budapest, and Warsaw) lie within 600 km distance from Katowice, the Silesia province capital. The main transport routes linking Poland with Western Europe run in all directions across this region.

The Electrical Low Pressure Impactor (ELPI, manufactured by DEKATI) was used to examine the number concentration and number size distribution. ELPI is used worldwide in continuous air quality monitoring for assessing PM size distribution and PM concentrations. It is often employed in examinations of dusts emitted from industrial sources or motor vehicles [14, 37–44].

The main components of ELPI are

- corona charger, used to charge particles with a known charge before they are collected in the impactor stages;
- low-pressure cascade impactor, used for size distribution of PM; it consists of 13 electrically insulated (PTFE isolators) stages, whose cut-off diameters gradually decrease; its first stage is the preselection stage;
- multichannel electrometer, used for measuring electric current that appears when particles are collected in different impactor stages; current intensity values constitute rough results obtained from the ELPI unit; they form the basis for calculating remaining output, that is, distribution of particle number, size, area, mass, and volume.

The course of the measurement process and specific instrument parameters were controlled with the computer and ELPI VI software.

Number size distribution was measured in real time for particles with aerodynamic diameters between 0.03 and 10 *μ*m. The measurements took place between January 1, 2010, and October 7, 2010. They were carried out in series lasting between six and fourteen days, depending on the concentration of total suspended particles (TSP) in the given period (necessary impactor clean-up—one day lasting technical break). Seventy measurement series were performed in total. ELPI was installed in an air-conditioned measurement container. The inlet with a sampling head for TSP was placed at the height of 4.5 m above the ground level.

## 3. Results and Discussion

The results are presented in several specific subsections focusing on the main questions that prompted this study.

### 3.1. Particle Number Concentrations

Descriptive statistics for sets of number concentrations (concentration is the amount of PM particles per cm^3^) of 13 PM fractions at the urban location site are given in Tables 1 and 2. The basic averaging time for results obtained with ELPI (moving average) was 1 minute. 1-minute concentrations were averaged to 1 hour (Table 1), whereas 24-hour concentrations were calculated on the basis of average 1-hour concentrations (Table 2). Average concentrations were calculated twice during the measurement period. The first calculation was based on 1-hour concentrations, while the second one was based on 24-hour concentrations. The results were averaged for three measurements ranges, that is, for the entire research period (January 1, 2010–October 7, 2010), winter (January 1, 2010–March 31, 2010, and October 1, 2010–October 7, 2010), and summer (April 1, 2010–September 30, 2010).

The total number concentration (sum of average concentrations of all fractions) for the entire research period (January 1, 2010–October 7, 2010) was comparable to sets of 1-hour (Table 1) and 24-hour concentrations (Table 2). It was 6227.76 cm^−3^ and 6063.5 cm^−3^, respectively. The discrepancy could result from short-term episodes of high particle concentrations in the air, which could have considerable impact on the value of the average 24-hour number concentration [15].

It is useful to compare the results obtained in this study with those obtained in similar studies around the world (Table 3). At first, it is worth mentioning that the differences in the observed number concentrations resulted, in a large degree, from different characteristics of the measurement sites, dissimilar local conditions, distinct periods of result averaging, and diverse measurement instruments [12].

In general, the total number concentration of PM in Zabrze was lower than number concentrations of ambient particles at the majority of measurement sites in Europe, with the exception of values recorded in Vienna, Prague, Hohenpeißenberg, and Budapest (Table 3). However, in winter the average number of particles in Zabrze was similar to the results obtained in other places.

The results listed in Table 3 suggested that PM number concentration in Zabrze is mainly influenced by primary emissions, whereas in other European regions, gas-to-particle conversion mostly affected on aerosol number. It was clearly visible in the Hohenpeißenberg research, where the higher particle number was observed in summer, when meteorological conditions were favorable to the formation of secondary aerosol particles. Moreover, it is a fact that mass concentrations of PM_2.5_ and PM_10_ in Zabrze have nearly the highest values in Europe [34, 45].

The number concentrations of PM generally decrease with increasing particle diameter [15]. Over 80% of the airborne particles in the urban air are in the ultrafine size range [21].

The measurements conducted in Zabrze revealed that 99% of airborne particles have diameters ≤1 *μ*m. PM_0.056_ constituted 44% of the total number concentration, whereas PM_0.156_ and PM_0.263_ made 62% and 79% of the total number concentration of PM (Table 1). Similar results were obtained at the urban site in Milan, Italy. The number concentrations of ultrafine and submicron particles constituted 78 ± 5% and 22 ± 5% of the total number concentration in winter and 79 ± 5% and 21 ± 5% of the total number concentration in summer, respectively [46].

The obtained results corroborated the fact that ultrafine particles had the largest share in the total PM number concentration observed in Zabrze. Consequently, this meant that the PM in Zabrze came mainly from combustion (fossil fuel, biomass, petrol, and gas) and transformations of gaseous precursors of PM.

It should be noted that ultrafine particles constitute a major threat for the local inhabitants and environment [3, 47–49]. For example, it was found that inhaled or instilled ultrafine PM particles induce pulmonary inflammation, oxidative stress, and distal organ involvement in animals [50–52]. Moreover, they can also induce or exacerbate pulmonary and cardiovascular diseases in humans, such as COPD and asthma in children and compromised adults [16, 52–54].

### 3.2. Seasonal Variation of Particle Number Concentrations

Measurements conducted at background site in Zabrze, which lasted for 9 months, revealed also distinct seasonal variation of particle number concentrations. In winter, the total average particle number in the air, whether for the averaged 1-hour or 24-hour results, was nearly twice as high as the analogous averaged value calculated for summer (Tables 1 and 2). It was 8797.87 and 4946.9 cm^−3^ (sets of 1-hour concentrations) and 8461.72 and 4915.07 cm^−3^ (sets of 24-hour concentrations) for winter and summer seasons, respectively. The visible seasonal variation was observed not only for the total number concentration but also for all PM fractions.

While analyzing seasonal changes in the total particle number concentrations, it was found that total PM number concentrations were 1.78 times higher in winter than in summer. However, while analyzing seasonal changes in the particle number within particular aerodynamic diameter ranges, it was observed that increases in particle number varied considerably and ranged from 1.22 to 4.29. The largest one occurred for diameters between 0.617 and 2.41 *μ*m (3.18–4.29). The lowest one occurred for fractions between 0.028 and 0.056 *μ*m (1.2–1.5)—Tables 1 and 2.

Generally, the maximum values observed for sets of 1-hour and 24-hour concentrations were also higher in winter, with the exception of the maximum number concentrations of the following fractions: 0.028–0.056 *μ*m, 0.056–0.095 *μ*m, 0.384–0.617 *μ*m, and 0.617–10.0 *μ*m. For particles from the 0.028–0.617 *μ*m range it was caused by intensive nucleation processes occurring in summer (higher solar radiation intensity and high relative air humidity). Coarser particles originated mainly from mechanical processes, particularly road and soil erosion, and from biogenic sources such as vegetation-derived primary bioaerosol (e.g., pollen, spores, and plant debris). Consequently, their higher concentrations were observed in a dry and warm period (summer).

A visible seasonal variation of PM number concentration was also observed in other European cities (e.g., see [10, 12, 15, 46]). For example, the total number concentration and number concentration of ultrafine (particles with diameter less than 100 nm) and submicron particles (particles with diameter between 100 and 1000 nm) in winter were nearly twice as high as summer concentrations, in measurements conducted at the urban site in Milan, Italy [46]. Similar observations were made at other measurement sites in Europe [10, 21, 60].

A more intensive impact from house heating in winter and meteorological conditions that influenced the boundary layer height (mixing layer) could be the reasons for the observed seasonal variation. During winter atmospheric conditions were significantly more stable, which resulted in the stagnation of pollutants and prevented their dispersion in the air [46, 61]. On the contrary in summer period the boundary layer is higher than in winter for the stronger convection induced by the solar radiation, resulting in a greater vertical dilution of the pollutants [46]. Moreover, in spring and summer higher concentration of aerosol precursor gases may allow photochemical reactions to produce condensable gases and subsequent nucleation and growth in urban [21]. It would result in increased particle number concentration for ultrafine particles. The study by Borsós et al. demonstrated that elevated PM number concentrations were mainly observed in summer months, particularly for ultrafine particles [12]. Number concentrations were also the highest in summer (3101 cm^−3^) and lowest in winter (1807 cm^−3^) at the rural background site in Hohenpeißenberg, Germany [15].

The enhanced concentrations in winter would be attributed especially to particulate emissions from domestic heating and power generation sector [60].

### 3.3. Number Size Distribution

**Figure 3:**
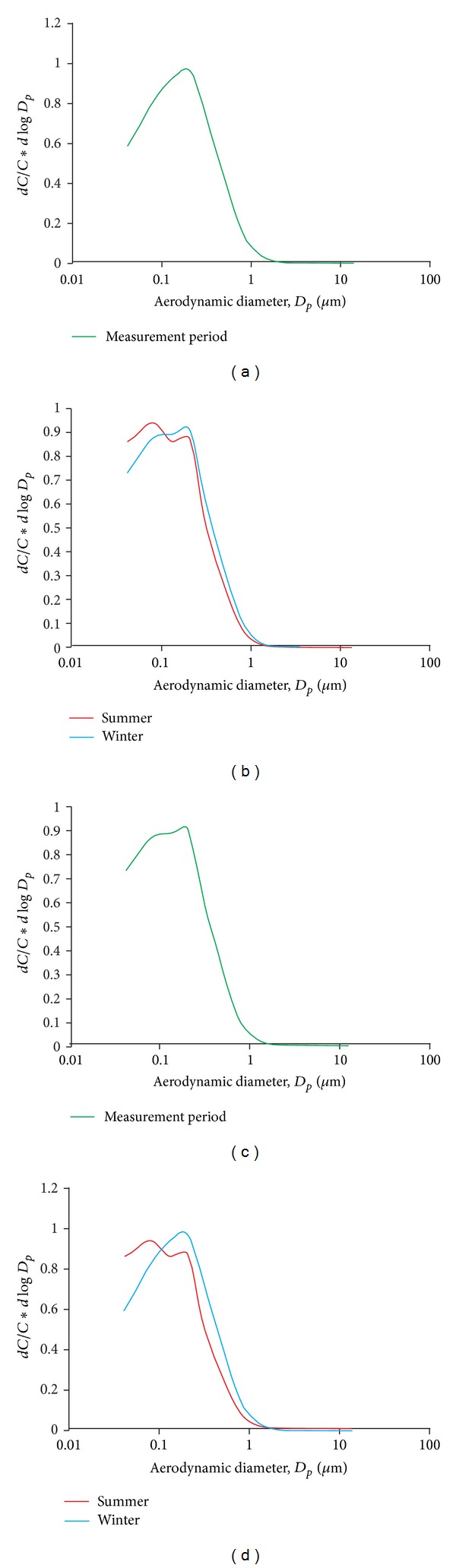
Number size distribution: (a) 1-hour concentrations, for entire measurement period (January 1, 2010–October 7, 2010); (b) 1-hour concentrations, in summer (April 1, 2010–September 30, 2010) and winter (January 1, 2010–March 31, 2010, and October 1, 2010–October 7, 2010); (c) 24-hour concentrations, for entire measurement period (January 1, 2010–October 7, 2010); (d) 24-hour concentrations, in summer (April 1, 2010–September 30, 2010) and winter (January 1, 2010–March 31, 2010, and October 1, 2010–October 7, 2010).

Figure 3 presents number size distribution for the discussed periods calculated on the basis of 1-hour and 24-hour concentrations. Many authors demonstrate [12, 46, 56, 62] that the number size distribution of PM is rather dynamic. It reflects the influence of emission sources as well as processes of PM particles formation, transformation, and transportation in the atmosphere.

Whether it was established on the basis of 1-hour or 24-hour concentrations (Figure 3), the number size distribution was unimodal within the entire measurement period in 2010. The maximum of number size distribution occurred for the 0.157–0.263 *μ*m fraction (Figures 3(a) and 3(c)). The number size distribution was bimodal in shorter time periods, namely, for 1-hour concentration distribution in winter and 24-hour concentration distribution in summer (the second distribution maximum for the 0.056–0.095 *μ*m fraction).

Similar results were obtained in Zabrze in 2008. The research concerned analyses of mass size distribution of PM [63]. The maxima of mass size distribution occurred for the 0.26–0.4 *μ*m fraction in summer and for the 0.17–0.26 *μ*m fraction both in winter and in the entire measurement period.

The distribution of ambient particles as a function of particle size, whether in urban or remote air, is typically characterised by three main modes [64]: the nucleation mode, the accumulation mode, and the coarse mode. The modes reflect the dominant processes giving rise to ambient PM [3]. Nucleation mode particles derive from chemical and physical processes, such as nucleation and condensation of supersaturated vapours produced by combustion. Accumulation mode particles mainly come from the growth of nucleation mode particles by coagulation, whereas most of coarse mode particles originate from anthropogenic and natural mechanical processes [65]. The coarse mode particles are usually primary particles generated by mechanical abrasion processes, but may contain other constituents as a result of coagulation and condensation processes [3].

The results showed that maxima of number size distribution fall into accumulation mode particles (with aerodynamic diameter between 100 nm and 1000 nm). Nucleation mode particles are short-lived (minutes to hours) and grow by coagulation or vapour adsorption to form the accumulation mode. Particles in this latter size range can remain suspended for several days since further growth is inefficient and gravitational settling and deposition are slow [3].

Observed seasonal variation of particle number concentration in Zabrze was linked with occurrence of additional maximum for nucleation mode particles (Figures 3(b) and 3(d)). Its presence might be related to the significant contribution of secondary products of precursor transformations in the total number of PM observed in Zabrze. The seasonal variation of nucleation episodes was related to local characteristics, such as solar radiation intensity, air temperature, relative humidity, biogenic emissions, wind speed, and the atmospheric boundary level height, as well as the PM concentration and PM size distribution [12, 66–68]. In winter period the occurrence of second maximum for nucleation mode particles might have resulted from combustion processes, much more intensive in cold months, which emit large quantities of gaseous precursors [11].

### 3.4. Daily Variation

**Figure 4:**
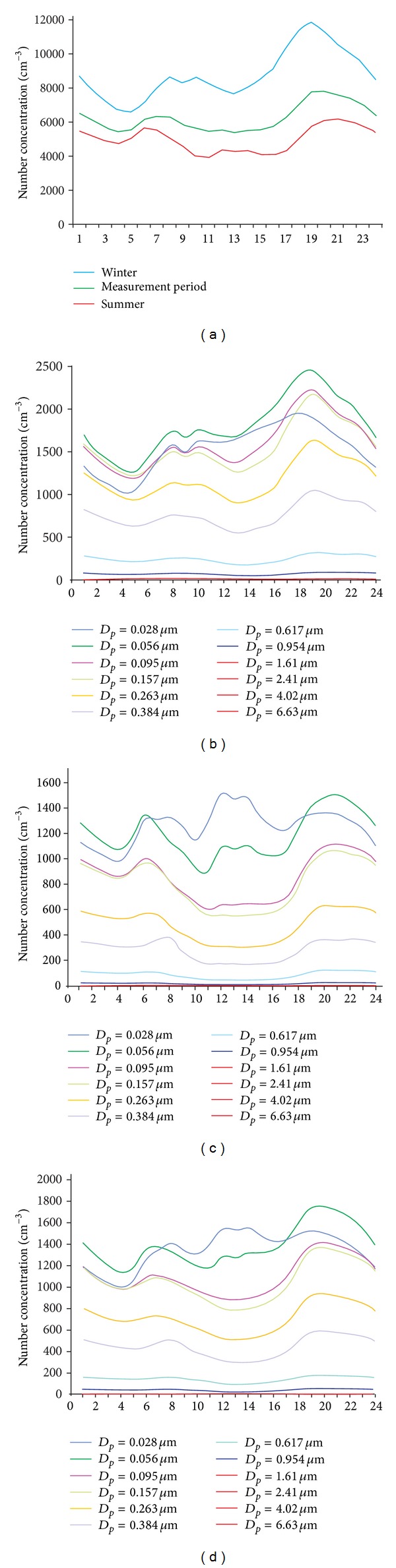
Changes in the total particle number 1-hour concentrations (a) and particle number of the 13 PM fractions; (b) 1-hour concentrations in winter (January 1, 2010–March 31, 2010, and October 1, 2010–October 7, 2010); (c) 1-hour concentrations in summer (April 1, 2010–September 30, 2010); (d) 1-hour concentrations entire measurement period (January 1, 2010–October 7, 2010), within 24-hour period.

The number concentration of PM was visibly variable within a short time period (few hours)—Figure 4. Generally, the most significant variation was observed for the finest particles.

In winter, the number concentration of PM increased in the morning (6.00–10.00). A visibly large increase was also observed in the late afternoon and evening. In summer, maximum particle concentrations occurred at a slightly earlier time (5.00–8.00) in the morning. The evening maximum was observed at a slightly later time than in winter.

The number size distribution of PM for averaged results at each hour, observed for the entire measurement period, was unimodal with the maximum for the range of 0.157–0.263 *μ*m. The second maximum (0.056–0.095 *μ*m) was observed for averaged results at each hour in two periods (18.00–23.00 and 5.00–8.00) in summer and early afternoon (12.00–14.00) in summer and in winter.

The daily patterns of particle number concentrations are interpreted in the light of the daily patterns of the emission sources and of the evolution of the main meteorological factors affecting the dispersion of atmospheric pollutants [46]. The variation in 1-hour concentrations within 24-hour period was similar in both measurement periods (summer and winter). However, for the summer, due to the less intensity of domestic heating emissions and to the different evolution of the boundary layer (earlier rise in the morning, higher height in the afternoon, and later fall in the evening), the concentration levels are quite different compared to the winter.

The morning and afternoon maxima of number size distribution of PM occurred both in winter and in summer, which could result from traffic emissions at rush hours [57]. It is noted that the mixing of the boundary layer is also increased during the early morning hours because of increasing sun radiation [10]. The number concentrations of all fractions decreased steadily from 10:00 PM towards noon. This feature is ascribed to the dilution of the emitted pollutants in an increasingly well-mixed boundary layer more than changes in traffic density [60]. The second maximum of number size distribution does not appear during the afternoon rush hours but shows up in the late evening. The shifts of the second peak into the late evening hours can be mainly explained by the effect of meteorology [12]. House heating, especially in winter season, could possibly contribute to the high PM number concentration observed at late evening hours [12, 22, 25]. The additional summer maximum of the number size distribution (occurring for the finest particles) in the early afternoon could be influenced by nucleation events. Such processes occur intensively in the presence of strong solar radiation [60, 67].

The daily variation of the PM number concentration at the urban site in Zabrze was similar to its variations observed in other European cities [10, 12, 23, 46, 60].

## 4. Conclusions

Particle number concentration and number size distribution of atmospheric particles in the aerodynamic diameter range from 0.028 to 10 *μ*m were determined in Zabrze at urban background location. Particle number concentration in thirteen size fractions and their diurnal and seasonal variations were derived and compared. The measurements embraced seasons typical for the Upper Silesian urban area (heating/nonheating ones, winter/summer), which differed in meteorological and emission conditions. The following facts were established.

1. Presence of 2 maxima (for ranges of 0.056–0.095 *μ*m and 0.157–0.263 *μ*m) of number size distribution of PM occurred in Zabrze.
2. Long-term measurements at urban background site in Zabrze revealed distinct seasonal patterns. The average particle number in winter was nearly twice as high as in summer, regardless of the averaging time (24 hours, 1 hour), as consequence of more frequent inversion situations and enhanced particulate emissions.
3. In comparison with summer, the largest increase in the particle number observed in winter concerned particles with diameters between 0.617 and 2.41 *μ*m, that is, fractions of the particles emitted from combustion processes.
4. In comparison with summer, the lowest increase in the particle number observed in winter concerned particles with diameters between 0.028 and 0.056 *μ*m, that is, particles from processes of gas transformations in precursors.
5. Measurements conducted in Zabrze revealed that most of the background particle number concentration was derived from submicron particles. PM_1_ constituted 99% of all particles observed in Zabrze.
6. Particle number concentrations were highly variable on a time scale of several hours. The variation in the average 1-hour particle number within 24 hours was similar for both seasons. The highest values of particle concentrations were observed for two periods, that is, in the morning (traffic rush hour, 7.00–10.00) and in the afternoon or late evening (traffic and house heating emissions). An additional maximum was observed in the early afternoon in summer. The particle size (0.028–0.056 *μ*m) proved that the maximum was related to the intensive formation of new PM particles from gas precursors at that time of day.
7. The diurnal cycles of particle number concentrations resemble the time-activity pattern of inhabitants, particularly in the pattern of road traffic flow, and are affected by meteorological circumstances. Traffic was found to be important source of ultrafine particles. Fossil fuel combustion from local sources and atmospheric nucleation contribute substantially. Diurnal variations of the intensities of these sources, particularly in road traffic, exhibit analogies for other urban areas.
8. In general, PM number concentration at the Zabrze urban background site was lower than number concentrations at the majority of measurement sites in Europe.

In order to be able to study urban aerosol dynamics and the possible health effects of aerosol particles long continuous data sets are needed. The present data is a good example of this kind of data set, which can be used for different purposes.

## Figures and Tables

**Table 1:** Descriptive statistics for sets of 1-hour (1 h) number concentrations of PM in Zabrze.

Impactor stage^a^	Period	Number of 1 h concentration^c^	Time coverage	Min. (1/cm^3^)	Max. (1/cm^3^)	Arithmetic mean (1/cm^3^)	Standard deviation (1/cm^3^)	25th percentile (1/cm^3^)	Median (1/cm^3^)	75th percentile (1/cm^3^)
0.028	MP^b^	5310	79.02	0	28384.85	1371.99	1237.34	560.46	1033.41	1771.27
	S^b^	3544	80.69	0	28384.85	1278.74	1203.52	523.62	949.54	1662.41
	W^b^	1766	75.86	31.08	8327.33	1559.14	1282.26	665.17	1197.88	1971.05

0.056	MP^b^	5310	79.02	0.88	16029.78	1393.02	1213.68	583.46	1038.97	1778.77
	S^b^	3544	80.69	0.88	16029.78	1183.3	945.08	535.59	928.68	1532.3
	W^b^	1766	75.86	52.86	11797.33	1813.88	1539.9	733.95	1353.75	2428.77

0.095	MP^b^	5310	79.02	0.11	11696.4	1095.46	1068.7	435.62	788.77	1364.62
	S^b^	3544	80.69	0.11	5890.98	839.54	645.6	385.89	656.22	1101.94
	W^b^	1766	75.86	40.42	11696.4	1609.04	1484.08	638.22	1160.83	2047.33

0.157	MP^b^	5310	79.02	0	10527.43	1048.02	1006.06	431.01	736.69	1294.71
	S^b^	3544	80.69	0	5792.17	796.79	599.59	390.39	620.32	1022.84
	W^b^	1766	75.86	36.22	10527.43	1552.18	1393.23	611.02	1141.64	1954.01

0.263	MP^b^	5310	79.02	0	7630.67	703.28	751.47	266.3	444.91	844.75
	S^b^	3544	80.69	0	4611.7	470.64	373.48	233.6	362.92	576.57
	W^b^	1766	75.86	23.28	7630.67	1170.15	1044.74	454.13	860.67	1495.18

0.384	MP^b^	5310	79.02	0	17493.35	437.58	561.78	147.61	260.37	519.61
	S^b^	3544	80.69	0	17493.35	278.9	414.94	125.48	199	321.45
	W^b^	1766	75.86	27.85	4991.05	756.01	671.94	308.15	551.11	931.84

0.617	MP^b^	5310	79.02	0	1650.12	134.19	173.97	35.18	69.42	161.02
	S^b^	3544	80.69	0	892.51	77.78	97.28	27.29	46.56	83.45
	W^b^	1766	75.86	10.61	1650.12	247.39	229.8	101.96	172.83	296.2

0.954	MP^b^	5310	79.02	0	539.66	35.09	53.9	5	13.4	44.19
	S^b^	3544	80.69	0	258.86	16.77	27.92	3.36	7.17	16.03
	W^b^	1766	75.86	0.33	539.66	71.85	71.75	25.75	50.53	88.06

1.61	MP^b^	5310	79.02	0	114.04	6.42	10.12	1.25	2.61	7.54
	S^b^	3544	80.69	0	47.36	3.07	4.48	0.94	1.64	3.02
	W^b^	1766	75.86	0.38	114.04	13.15	14.14	4.41	8.68	15.64

2.41	MP^b^	5310	79.02	0	40.25	2.15	3.42	0.52	0.99	2.34
	S^b^	3544	80.69	0	14.24	1.09	1.36	0.4	0.68	1.17
	W^b^	1766	75.86	0.14	40.25	4.27	4.96	1.46	2.59	4.96

4.02	MP^b^	5310	79.02	0	5.71	0.3	0.5	0.07	0.15	0.32
	S^b^	3544	80.69	0	2.79	0.17	0.23	0.04	0.1	0.19
	W^b^	1766	75.86	0	5.71	0.57	0.74	0.18	0.3	0.63

6.63	MP^b^	5310	79.02	0	13.47	0.16	0.31	0.04	0.09	0.17
	S^b^	3544	80.69	0	13.47	0.11	0.3	0.03	0.06	0.12
	W^b^	1766	75.86	0	2.28	0.25	0.31	0.09	0.13	0.28

^a^Cut-off diameter of the impactor stage to which the line refers.

^b^MP: measurement period: January 1, 2010–October 7, 2010; S: summer, April 1, 2010–September 30, 2010; W: winter: January 1, 2010–March 31, 2010, and October 1, 2010–October 7, 2010.

^c^Arithmetic means for hours with at least 45 1-minute measurements.

**Table 2:** Descriptive statistics for sets of 24-hour (24 h) number concentrations of PM in Zabrze.

Impactor stage^a^	Period	Number of 24 h concentration^c^	Time coverage	Min. (1/cm^3^)	Max. (1/cm^3^)	Arithmetic mean (1/cm^3^)	Standard deviation (1/cm^3^)	25th percentile (1/cm^3^)	Median (1/cm^3^)	75th percentile (1/cm^3^)
0.028	MP^b^	280	75	189.46	4543.99	1340.15	858.22	671.21	1097.38	1788.22
	S^b^	183	77.6	189.46	3850.79	1274.52	792.78	636.67	1042.03	1760
	W^b^	97	70.1	190.91	4543.99	1477.2	966.62	811.82	1271.66	1856.99

0.056	MP^b^	280	75	226.8	5367.99	1351.73	878.88	758.41	1086.64	1761.2
	S^b^	183	77.6	232.67	3255.41	1173.78	622.52	704.25	1010.49	1539.38
	W^b^	97	70.1	226.8	5367.99	1723.32	1171.32	920.49	1352.37	2135.21

0.095	MP^b^	280	75	161.87	6895.54	1066.5	874.03	571.08	850.56	1248.42
	S^b^	183	77.6	161.87	2265.24	832.06	425.27	491.44	740.19	1035.76
	W^b^	97	70.1	325.96	6895.54	1556.07	1275.57	775.15	1145.23	1937.02

0.157	MP^b^	280	75	158.58	5903.64	1019.66	775.49	559.98	822.47	1191.85
	S^b^	183	77.6	158.58	2004.65	791.1	371.09	518.55	734.06	995.12
	W^b^	97	70.1	208.16	5903.64	1496.93	1110.31	761.42	1174.66	1823.83

0.263	MP^b^	280	75	110.36	3797.77	684.9	584.62	333.68	492.53	820.03
	S^b^	183	77.6	110.36	1398.62	467.36	234.28	300.66	419.64	584.64
	W^b^	97	70.1	128.07	3797.77	1139.17	797.3	595.8	945.58	1282.53

0.384	MP^b^	280	75	67.26	2287.28	425.27	402.66	184.93	292.92	538.94
	S^b^	183	77.6	67.26	2258.32	277.14	226.82	152.12	215.46	311.86
	W^b^	97	70.1	169.53	2287.28	734.61	501.75	394.54	564.37	836.56

0.617	MP^b^	280	75	10.75	884.6	131.72	141.73	43.94	73.26	172.58
	S^b^	183	77.6	10.75	388.74	77.96	72.55	32.83	51.79	85.39
	W^b^	97	70.1	54.44	884.6	243.99	180.02	128.06	183.72	272.84

0.954	MP^b^	280	75	0.51	289.42	34.58	44.56	6.3	14.27	49.16
	S^b^	183	77.6	0.51	102.71	16.67	20.46	4.74	8.21	16.79
	W^b^	97	70.1	13.22	289.42	71.96	56.47	34.23	54.46	86.63

1.61	MP^b^	280	75	0.1	66.64	6.39	8.7	1.49	2.8	7.94
	S^b^	183	77.6	0.1	19.52	3.09	3.4	1.22	1.84	2.81
	W^b^	97	70.1	2.89	66.64	13.3	11.79	5.67	9.07	16.28

2.41	MP^b^	280	75	0.08	25	2.14	2.99	0.61	1.14	2.48
	S^b^	183	77.6	0.08	6.28	1.1	1.04	0.47	0.75	1.15
	W^b^	97	70.1	0.96	25	4.33	4.28	1.85	2.78	5.27

4.02	MP^b^	280	75	0	3.69	0.3	0.45	0.08	0.18	0.34
	S^b^	183	77.6	0	0.98	0.17	0.18	0.06	0.11	0.21
	W^b^	97	70.1	0.13	3.69	0.58	0.66	0.21	0.34	0.63

6.63	MP^b^	280	75	0	1.89	0.16	0.22	0.05	0.1	0.18
	S^b^	183	77.6	0	1.89	0.12	0.18	0.04	0.07	0.13
	W^b^	97	70.1	0.04	1.48	0.26	0.27	0.1	0.15	0.28

^a^Cut-off diameter of the impactor stage to which the line refers.

^b^MP: measurement period: January 1, 2010–October 7, 2010; S: summer April, 1, 2010–September 30, 2010; W: winter: January 1, 2010–March 31, 2010, and October 1, 2010–October 7, 2010.

^c^Arithmetic means for hours with at least 45 1-minute measurements.

**Table 3:** Average number concentrations of PM observed at various urban background sites in the world.

Country	City, measurement period	Number concentration (cm^−3^)	Reference
**Poland**	**Zabrze, Jan.–Oct.** 2010^1^	**Whole period: **6.23 · 10^3^ **Summer: **4.95 · 10^3^ **Winter: **8.80 · 10^3^	**The following study**

Austria	Vienna	8.0 · 10^3^	[12]

Czech Republic	Prague	7.3 · 10^3^	[12]

Finland	Helsinki, 2001–2003	Weekdays: 1.1 · 10^4^ Weekends: 0.69 · 10^4^	[10]

France	Marseille, 2002-2003	1 · 10^4^	[55]

Germany	Augsburg, 2001–2003	Weekdays: 1 · 10^4^ Weekends: 0.82 · 10^4^	[10]
	Hohenpeißenberg, 2003-2004	Summer: 3.10 · 10^3^ Winter: 1.81 · 10^3^	[15]

Greece	Athens, 2002-2003	1 · 10^4^	[55]

Hungary	Budapest	10.6 · 10^3^	[12]

Italy	Rome, 2001–2003	4.3 · 10^4^	[10]
	Milan, Sept 2003–Aug 2004	Cold season: 2.5 · 10^4^ Warm season: 1.3 · 10^4^	[46]

The Netherlands	Utrecht, Oct.-Nov. 2008	Street location: 3.86 · 10^4^ Suburban background: 133 · 10^4^ City background: 141 · 10^4^	[56]

Spain	Barcelona, 2001–2003	Weekdays: 3.9 · 10^4^ Weekends: 2.76 · 10^4^	[10]
	Barcelona, 2003-2004	2.3 · 10^4^	[10]

Sweden	Stockholm, 2001–2003	Weekdays: 1.0 · 10^4^ Weekends: 0.8 · 10^4^	[10]

Switzerland	Zurich, 2001-2002	4.7 · 10^4^	[20]

UK	London, 2004-2005	5 · 10^4^	[57]

Australia	Brisbane	1.5 · 10^4^	[58]

USA	Atlanta, 1998-1999	9.7 · 10^4^	[59]

^1^Values given in the table were obtained on the basis of average 1-hour concentrations (Table 1).
